# Changing to improve? Organizational change and change-oriented leadership in hospitals

**DOI:** 10.1108/JHOM-09-2019-0280

**Published:** 2020-08-25

**Authors:** Olaug Øygarden, Espen Olsen, Aslaug Mikkelsen

**Affiliations:** 1 University of Stavanger Business School , Stavanger, Norway; 2 NORCE Norwegian Research Centre , Stavanger, Norway

**Keywords:** Physicians, Hospital management, Organizational change, Participation in decision-making, Change-oriented leadership, Hospital service quality

## Abstract

**Purpose:**

This paper aims to fill gaps in one’s knowledge of the impact of organizational change on two outcomes relevant to hospital service quality (performance obstacles and physician job satisfaction) and in one’s knowledge of the role of middle manager change-oriented leadership in relation to the same outcomes. Further, the authors aim to identify how physician participation in decision-making is impacted by organizational change and change-oriented leadership, as well as how it mediates the relationships between these two variables, performance obstacles and job satisfaction.

**Design/methodology/approach:**

The study adopted a cross-sectional survey design including data from Norwegian hospital physicians ( *N* = 556). A hypothetical model was developed based on existing theory, confirmatory factor analysis was carried out in order to ensure the validity of measurement concepts, and the structural model was estimated using structural equation modelling.

**Findings:**

The organizational changes in question were positively related to performance obstacles both directly and indirectly through participation in decision-making. Organizational change was also negatively related to job satisfaction, both directly and indirectly. Change-oriented leadership was negatively related to performance obstacles, but only indirectly through participation in decision-making, whereas it was positively related to job satisfaction both directly and indirectly.

**Originality/value:**

The authors developed a theoretical model based on existing theory, but to their knowledge no other studies have tested these exact relationships within one model. These findings offer insights relevant to current and ongoing developments in the healthcare field and to the question of how hospitals may deal with continuous changes in ways that could contribute positively towards outcomes relevant to service quality.

## Introduction

Over the past four decades, hospitals internationally have undergone dramatic changes in their funding, organization, management, service delivery and regulation as a result of a multitude of public sector reforms. These reforms have largely been categorized as inspired by new public management (NPM), meaning that they have been motivated by a desire to improve service efficiency and effectiveness, responsiveness to the public and managerial accountability and to reduce public spending ( Christensen and Lægreid, 2011b ). A common theme in the understanding of NPM reforms is that managerial and economic principles have gained in importance vis-a-vis bureaucratic and professional principles ( Byrkjeflot and Kragh Jespersen, 2014 ; Hood, 1991 ). While the contents of the most recent public sector reforms and policies are sometimes labelled as post-NPM, implying a focus on reintegration of systems which have become fragmented as a result of NPM efforts, these more current initiatives have not diminished the importance of managerial and economic principles ( Christensen and Lægreid, 2011a ).

Norwegian hospitals have been the target of several NPM- and post-NPM-inspired reforms and policies. These reforms and policies have introduced stronger management functions, market instruments and increased transparency ( Byrkjeflot, 2011 ). As hospitals strive to meet new demands posed by reforms and societal expectations, organizational changes of different magnitude and at different organizational levels have become the normal situation ( Bernstrøm and Kjekshus, 2015 ). The totality is a situation in which hospital employees constantly have to adapt to new organizational structures and work arrangements.

The aim of this paper is threefold. First, organizational changes may be initiated for a wide variety of reasons, and not all planned organizational change in hospitals is directed primarily at improving the quality of services that hospitals provide. This paper, however, assumes that improving quality is in fact a central, strategic goal for policymakers and for decision-makers at the hospital organization level, and as such also a central goal when it comes to organizational changes in these organizations. Yet, it has been difficult to identify clear and positive performance effects of the organizational changes that flow from new policies and reforms ( Braithwaite *et al.* , 2016 ). In other words, we do not know whether changes presumably aiming to improve, and certainly not aiming to deteriorate, hospital services actually contribute to improvement. This paper aims to contribute towards filling this gap in our knowledge of the effects of organizational changes in hospitals. It does so by focussing on one organizational as well as one employee outcome, both of which are vital to hospital service quality, namely the resources and organizational structures surrounding patient treatment, and the job satisfaction of those providing treatment ( Casalino and Crosson, 2015 ; Carayon *et al.* , 2006 ).

Second, we know that good management matters in achieving high-quality hospital care, but knowledge regarding which practices work to what ends and how they work is still incomplete ( Lega *et al.* , 2013 ). We further know that hospital middle managers hold positions in which they are expected to channel, mediate and translate continuous organizational change initiatives to their staff at the operative level, but also that this is a very challenging role to fulfil ( Birken *et al.* , 2012 ; Williamsson *et al.* , 2016 ). This paper focusses on one specific leadership style which we believe to be relevant in a context characterized by continuous organizational change, namely change-oriented leadership ( Yukl, 1999 ) and its relationship to the two quality-related outcome variables. Change-oriented leadership is not among the most commonly studied leadership concepts within healthcare organizations ( Gilmartin and D'Aunno, 2007 ), and our knowledge of how it might work in a hospital setting is therefore incomplete. By focussing on this leadership style, we aim to explore whether it is in fact a set of middle manager leadership activities that may be beneficial in terms of improving hospital service quality and to contribute towards the still incomplete knowledge of which practices work, to what ends and how they work.

Third, the literature on health system improvement highlights a need for physicians to be engaged in organizational decisions in order for positive change to materialize ( Spurgeon *et al.* , 2008 ), and physician participation in decision-making is a way of ensuring such engagement. NPM-inspired reforms have transformed and possibly diminished the power and influence of the medical profession ( Byrkjeflot, 2011 ; Noordegraaf and Steijn, 2014 ). Research following Norwegian hospital reforms has shown that physicians as a professional group have increasingly had to compete with other groups for influence in decision-making at the policy level as well as in the management of hospitals ( Byrkjeflot, 2011 ). However, the impact of current organizational changes on the opportunities for hospital physicians who are *not* in management positions to influence decisions regarding success criteria, goals, actions and other aspects of their work has received less attention. There is also a need for more empirical research on how the medical engagement that we know is needed, is fostered and how it contributes to improvements ( Denis and Baker, 2015 ). Our final aim is therefore to identify how physician participation in decision-making is impacted by organizational change and change-oriented leadership and how it mediates the relationships between these two variables, performance obstacles and job satisfaction.

Hospitals are complex organizations. One way of describing this complexity is through the concept of different worlds ( Glouberman and Mintzberg, 2001 ), each of which may be ruled by different logics ( Reay and Hinings, 2005 ) and in which central actors hold differing identities or mindsets ( Andersson, 2015 ; Bååthe and Norbäck, 2013 ). For the sake of this paper, the most relevant worlds are the control world, inhabited by managers who hold managerial identities and are primarily guided by a business-like logic, and the cure world, inhabited by physicians who hold physician identities and are guided by a primarily medical professional logic ( Reay and Hinings, 2005 ). There is often little integration between these separate worlds that exist within each hospital organization ( Glouberman and Mintzberg, 2001 ), and organizational change may be initiated in and take place in each of them according to the priorities of the actors within them.

In this paper, we focus on organizational changes which are largely initiated from the control world, but also have a significant presence in and impact on the cure world. We do not focus on purely cure world changes, which could be changes to medical protocols or the introduction of new medical technology initiated by the professionals themselves. These changes are clearly also frequent and add to the totality of a complex and continuously changing context. However, changes to management, organizational structures and overall goals and strategies in hospitals also shape the work environment of operative level, cure world physicians. While focussing only on changes mainly connected to the control world means we are not able to capture the full complexity of organizational changes in hospitals, we believe it is also of value to clearly define, operationalize and study this one type of change. Many of these changes are guided by a business-like logic as opposed to the previously dominant professional logic ( Byrkjeflot, 2011 ). The introduction of business-like logic organizational structures and practices has resulted in increased managerial control, a focus on accountability, efficient use of resources, quantification of patient throughput, cost reduction, performance management, standardization of care, quality and customer orientation and customer satisfaction ( Reay and Hinings, 2009 ; Arman *et al.* , 2014 ). Organizational changes that involve transformation of organizational goals, beliefs and norms are referred to as divergent ( Battilana *et al.* , 2009 ). Such changes are often conflictual, and physicians are particularly prone to disagree with and oppose them ( Martinussen and Magnussen, 2011 ). Assuming that current organizational changes to management, organizational structures and overall goals and strategies are often divergent to the professional logic of physicians and that hospitals are continuously changing organizations, this study asks the following research question:

How are frequent organizational changes in hospitals and middle manager change-oriented leadership related to organizational and employee outcomes relevant to hospital service quality, and how are these relationships mediated by physician participation in decision-making?

## Theoretical framework

In this section, we first present arguments for the relevance of performance obstacles and job satisfaction as outcomes in relation to hospital service quality and then present the theoretical background for the hypotheses that make up our theoretical model.

### Performance obstacles

Our theoretical model contains two outcome variables, one representing organization of patient treatment and the other the well-being of hospital physicians. We begin by defining and explicating the importance of the first variable.

The quality of care provided by hospitals is dependent on a work system that enhances and facilitates the work performed by healthcare professionals. Work systems in hospitals, as modelled by Carayon *et al.* (2006) , consist of persons, tasks, tools and technologies, the physical environment and organizational conditions. If crucial elements of the system are missing or inadequately designed, service quality may suffer as the system hinders rather than helps physicians perform their job.

The national system of quality indicators defines the system elements of sufficient and functioning medical, ICT and other types of equipment, sufficient and competent staff and an organization of work that ensures coordination, collaboration and communication as structural quality indicators ( NDH, 2019 ). Accessibility of supplies and technology is vital to modern medicine ( Carayon *et al.* , 2006 ). There is also wide support in health services research for the importance of staffing adequacy in terms of both numbers and competence for quality of care ( Aiken *et al.* , 2002 ). The work system perspective considers the absence or suboptimal functioning of these elements as performance obstacles, defined as “the work system design characteristics that inhibit performance and are closely associated with the immediate work setting” ( Peters and O'Connor, 1988 ). In line with the national quality indicator system and the work system perspective, we consider performance obstacles to be a relevant measure of the service quality that a hospital is able to offer and believe it is important to understand how the frequency of organizational change is related to their prevalence.

### Job satisfaction

Job satisfaction is defined as “a pleasurable or positive emotional state resulting from the appraisal of one's job or job experiences” ( Locke, 1976 ). A recent panel study supports the hypothesis that there is a causal relationship running from job satisfaction to workplace performance ( Bryson *et al.* , 2017 ). This relationship may be explained by mechanisms such as increased energy and effort due to better general health following positive emotions or increased problem-solving skills due to improved cognitive abilities ( Diener and Chan, 2011 ; Fredrickson, 2001 ; Lin *et al.* , 2014 ).

Physicians have, in comparison to other less professionalized groups, been found to identify primarily with their professional colleagues and relatively less with the organization in which they perform their work ( Andersson, 2015 ; Johansen and Gjerberg, 2009 ). Their job satisfaction or dissatisfaction is therefore also believed to be related directly to their work ( Cassalino and Crosson, 2015 ). Earlier research indicates that job dissatisfaction has a negative impact on patients and service quality. Several mechanisms are suggested in this literature, including reduced cognitive capacity, concentration, effort, empathy and professionalism ( Casalino and Crosson, 2015 ; Firth-Cozens, 2001 ; Williams and Skinner, 2003 ). Based on the evidence of job satisfaction's importance to employees and their performance in general, and for physicians specifically, we believe it is indeed a vital element in ensuring that hospitals are able to provide the high quality and efficient care that health policies, reforms and changes at the organizational level aim to achieve. We intend to add to the knowledge of how such changes actually relate to physician job satisfaction.

### Organizational change and job demands

Reorganizing, changing management or introducing new overall goals and strategies may reduce the prevalence of performance obstacles if the new organizational structures and practices are wisely designed and implementation processes are successful. However, many of the ongoing changes in hospitals are motivated fully or partially by increasing resource efficiency (i.e. cut costs) and may therefore increase the prevalence of performance obstacles as resources diminish. Further, organizational change may also represent a job demand that negatively impacts hospital staff.

The job demands–resources (JD-R) model proposes that a wide variety of job characteristics are determinants of employee well-being and performance ( Bakker and Demerouti, 2007 ). The JD-R model posits that job demands, such as high work pressure, emotional demands or role ambiguity, negatively impact employee health and well-being, whereas job resources such as social support, performance feedback and autonomy may spark a motivational process leading to job-related learning, work engagement and organizational commitment.

Job demands are defined as “those physical, social or organizational aspects of the job that require sustained physical or mental effort and are therefore associated with certain physiological and psychological costs” ( Demerouti *et al.* , 2001 ). Organizational change can both increase existing and introduce new demands ( Smollan, 2017 ), as well as represent a demand in and of itself. Schaufeli (2015) clusters job demands into categories of qualitative, quantitative and organizational demands. Qualitative job demands include mental job demands, meaning the attention and concentration that the work requires. Frequent change in, for instance, organizational structures stemming from reorganizations requires constantly adapting to new ways of working, which may increase these mental demands. Quantitative demands include work overload and perceptions of the pace of change. In the hospital context, where many change efforts aim for increased efficiency, it is reasonable to assume that work overload may become more prevalent as a result of organizational change. Perceiving that changes are happening too fast, as may be the case if the frequency of change is high, is also regarded as a job demand.

The organizational changes measured in this study may be a demand which, in line with the JD-R model, leads to lower work engagement, defined as “a positive and fulfilling work-related state of mind characterized by vigour, dedication and absorption” ( Bakker *et al.* , 2008 ). Work engagement fosters extra-role performance ( Schaufeli and Salanova, 2014 ). It may therefore contribute to reducing the prevalence of performance obstacles, as engaged employees are more likely to go the extra mile and contribute to finding workarounds that enable patient treatment despite the existence of performance obstacles or solutions to eliminate performance obstacles. Reduced work engagement as a result of frequent organizational change may, conversely, lead to less such extra-role performance and to the persistence of performance obstacles. If organizational change is indeed a job demand, it may also contribute to negative emotions for employees, which narrows cognitive skills and the employees' ability to come up with workarounds and solutions ( Fredrickson, 2001 ). Our first hypothesis is that:

H1.
Organizational change will be positively related to performance obstacles.

Research testing the JD-R model has found that as job demands increase and job resources decrease, job satisfaction decreases as a result of maladaptive coping ( Alarcon, 2011 ). The category of organizational job demands also includes not agreeing with changes. If employees do not agree with a certain change effort, the organizational change *itself* is a demand. We know from previous research that Norwegian physicians have resisted NPM-inspired reforms and that they do not believe stated goals such as equality of access to care, medical quality and hospital productivity have been furthered by them. This belief is most strongly held by physicians who are not in management positions ( Martinussen *et al.* , 2017 ). Divergent changes to management, organizational structures and overall goals and strategies may therefore be considered as organizational job demands for this group. Given that physician job satisfaction is believed to be directly related to their work, changes that are perceived as limiting their opportunities to perform the work according to their preferred professional standards may therefore contribute to lower job satisfaction.

There is empirical support in the literature on organizational change in healthcare for expecting change to impact employees negatively in terms of physical and mental health ( Westgaard and Winkel, 2011 ; Bernstrøm and Kjekshus, 2015 ). This is particularly true when the changes are related to management, organizational structures, overall goals and strategies as opposed to merely technological or related to new modes of patient treatment. Regarding job satisfaction, a systematic review of previous studies concludes that the results are mixed ( Westgaard and Winkel, 2011 ; Grønstad *et al.* , 2019 ). Given our understanding of the changes included in our data as often divergent to the medical professional logic, we believe that higher frequencies of organizational change will be a job demand related to lower job satisfaction for physicians. We therefore hypothesize that:

H2.
Organizational change will be negatively related to job satisfaction.

### Change-oriented leadership

Leadership can be defined as “the process of influencing others to understand and agree about what needs to be done and how to do it, and the process of facilitating individual and collective efforts to accomplish shared objectives” ( Yukl, 2013 ). Following this definition, leadership is an activity or a set of behaviours. It is not exclusively tied to top executive positions, but may be performed by a wide range of actors who are in positions that allow them to influence other actors. Many theories on leadership styles make a distinction between leaders who primarily focus on production and work tasks and leaders who focus on staff relationships ( Borgmann *et al.* , 2016 ). Recognising a need to further elaborate the task–relations dichotomy of leadership behaviours, Yukl argues that it is important to distinguish between task-, relations- and change-oriented behaviours, because all of these three categories and the leadership tasks contribute to understanding effective leadership ( 1999 , Yukl *et al.* , 2002 , 2019 ). Task-oriented behaviours are primarily concerned with the efficient and reliable accomplishment of tasks, and relations-oriented behaviours with increasing mutual trust, cooperation and employee identification with the team or organization. Change-oriented leadership behaviours include monitoring and interpreting the environment, envisioning new possibilities for the organization, explaining the need for change, suggesting new and creative solutions and experimenting with new approaches for achieving objectives, taking a long-term perspective on problems and opportunities and negotiating for support from other actors on behalf of the department.

The leadership behaviours of interest in this study are performed by hospital middle managers. These managers are responsible for organizing staff and patient treatment in individual departments. This leadership role is considered as particularly challenging, due to the high complexity of actors, competencies, interests and authority relations that characterize hospitals ( Denis *et al.* , 2010 ). As actors holding managerial responsibilities while also being located at the operative lever, they hold a position balancing between and connecting the control and cure worlds and may perform both managerial and professional duties and forms of leadership. In this study, we are mainly interested in their managerial leadership and the ways in which they act on and lead their staff in the context of the specific organizational changes studied. While hospital middle managers are in charge of several processes in their respective departments, they are usually not the instigators of changes to management, organizational structures or overall goals and strategies. These types of changes more often stem from the levels above the departments, such as hospital top leadership, regional health authorities or national health policy. The leadership behaviours of middle managers may, however, still be more or less change-oriented. Their leadership role in terms of these particular types of organizational change is largely to act as mediators between higher levels and department-level employees ( Birken *et al.* , 2012 ).

In continuously changing contexts, leader behaviour and attitudes may be crucial to the way in which employees perceive, accept and are affected by change ( Sanchez-Burks and Huy, 2009 ). Change-oriented leadership may intuitively be associated mainly with leader initiation of a large number of changes. However, it is rather a leadership style characterized by being attuned and adaptive to the environment, explaining the need for change, finding ways of working at the operative level that may contribute to achieving new objectives and being skilled at processes of implementing changes. Exhibiting these leadership behaviours may be a way for middle managers to fulfil the role of change mediator for their employees. The source of stress related to rapidly changing work environments (in addition to the increased job demands outlined earlier) has also been found to be uncertainty and perceptions of poor change processes characterized by lack of consultation, information and management support ( Brown *et al.* , 2006 ; Smollan, 2017 ) and affective resistance to change ( Rafferty and Jimmieson, 2016 ). A change-oriented middle manager, who clearly communicates the reason for and content of change, may buffer the negative effects of continuously changing hospital environments by providing information in a convincing manner, thereby contributing to the employees' individual sensemaking ( Rouleau, 2005 ; Jimmieson *et al.* , 2004 ) and increased understanding of why new demands are introduced ( Bakker and Demerouti, 2007 ).

Change-oriented leadership has also been found to have a significant, although small, effect on performance ( Borgmann *et al.* , 2016 ). In the case of performance obstacles, change-oriented leaders may be effective in reducing their prevalence due to their ability to search for and suggest new solutions to department-level problems. Compared with task- and relations-oriented leadership, it has the largest (and a positive) influence on job satisfaction ( Borgmann *et al.* , 2016 ). We therefore hypothesize that:

H3.
Change-oriented leadership will be negatively related to performance obstacles.

H4.
Change-oriented leadership will be positively related to job satisfaction.

### Participation in decision-making

Based on the traditional centrality of autonomy and control for the medical profession, and the divergent nature of organizational changes inspired by NPM, we are particularly interested in the role played by physician participation in decision-making in mediating the impact of frequent organizational change. Job resources are defined as those physical, psychological, social or organizational aspects of the job that may be functional in achieving work goals, reduce job demands and their associated costs or stimulate personal growth and development ( Demerouti *et al.* , 2001 ). They are important tools in dealing with job demands and have a motivational potential but are also important in and of themselves. In the JD-R framework, job control is included as a job resource located at the level of the organization of work ( Bakker and Demerouti, 2007 ), and it includes not only autonomy over immediate tasks and time constraints but also participation in decision-making ( Alarcon, 2011 ). Job control has consistently been found to be an important job resource for fostering motivation and engagement. Job resources may also fuel job satisfaction ( Sousa-Poza and Sousa-Poza, 2000 ).

Participation in decision-making and having the opportunity to influence how work is performed is a highly valued resource for physicians. This means that we can expect it to be positively related to job satisfaction particularly for this group, and it also points to the importance of exploring how frequent organizational change experienced by physicians is related to this specific job resource. According to the conservation of resources theory of stress, which is a foundation of the JD-R model, “individuals strive to obtain, retain, foster, and protect those things they centrally value” ( Hobfoll *et al.* , 2018 ). If key resources are either threatened with loss, actually lost or significant effort fails to deliver expected resources, individuals will experience stress. Generally speaking, organizational change can be experienced as a threat since it poses a risk of losing valued resources such as status, income or comfort ( Dent and Goldberg, 1999 , Van Den Heuvel *et al.* , 2013 ). Organizational changes that take place in the wider context of a shift away from physician autonomy and self-regulation may lead to actual loss of the resource of participation in decision-making, or at least be perceived as a threat to this valued resource, leading to lower levels of well-being at work, lower engagement and negative emotions.

In addition to being a job resource which may contribute to reducing the prevalence of performance obstacles via employee motivation, work engagement and positive emotions as explicated earlier, physician participation in decision-making is also an aspect of what is referred to as medical engagement ( Spurgeon *et al.* , 2008 ). This form of engagement is conceptually distinct from work engagement and implies, among other things, the “active and positive contribution of doctors within their normal working roles to maintaining and enhancing the performance of the organization (…)” ( Spurgeon *et al.* , 2008 ). Medical engagement may serve to distribute decision-making to a wider set of actors, thereby allowing a more diverse set of expertise and skills to contribute to problem-solving ( Denis and Baker, 2015 ). Allowing decision latitude for physicians who are not in formal management positions may therefore serve to decrease the prevalence of performance obstacles.

The literature on change-oriented leadership outcomes does not clearly define the role of employee autonomy as a mediator ( Borgmann *et al.* , 2016 ). Change-oriented leadership may be positively related to autonomy because change-oriented leaders solicit the advice of employees in finding new solutions and facilitate participatory change processes leading to more employee involvement ( Bryson *et al.* , 2013 ). This can lead to an experience of participation in decision-making in the work setting for employees.

We hypothesize the following:

H5.
Employee participation in decision-making will mediate the influence organizational change and change-oriented leadership have on performance obstacles and job satisfaction.

H5a.
Organizational change will be negatively related to participation in decision-making.

H5b.
Change-oriented leadership will be positively related to participation in decision-making.

H5c.
Participation in decision-making will be negatively related to performance obstacles.

H5d.
Participation in decision-making will be positively related to job satisfaction.

### Conceptual framework

**Figure 1:**
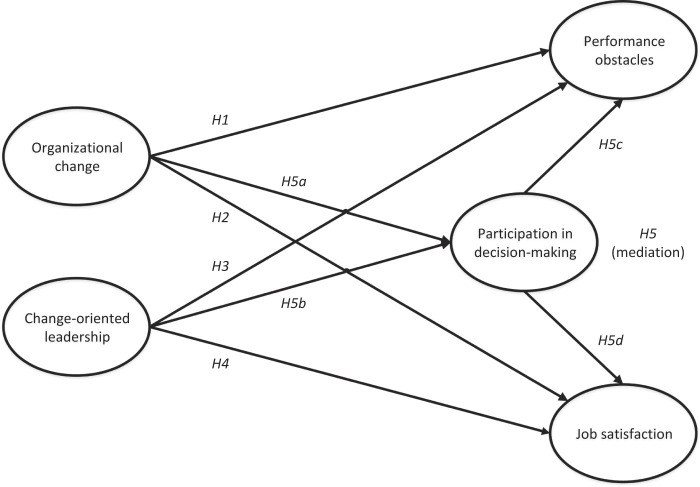
Theoretical model and hypotheses underlying this study

Our hypotheses are illustrated in Figure 1 .

## Methods

### Research design, survey and participants

We collected the data for this study from four Norwegian hospitals. The study adopted a cross-sectional web-based survey design distributed via an internal web application to all of the health authority's employees. The survey consisted of a range of validated questions tailored to (1) carrying out a work environment survey commissioned by the regional health authority and (2) collect research data. The aim of the present paper was to study physicians, and only physicians who do not hold management positions were therefore included ( *N* = 556). The response rate for physicians was 24.5%. Respondents gave their informed consent by turning in the questionnaire, and all survey responses were anonymous. The Norwegian Centre for Research Data and the relevant hospital committees approved the research.

### Measures

*Organizational change* was measured using three items asking respondents to rate the extent to which various events (changes) had affected their organization within the past 12 months ( Baron and Neuman, 1996 ). The events included were (1) changes in management, (2) reorganization and (3) establishment of new overall goals and strategies. The items were measured on a four-point scale ranging from “not at all” (1) to “to a great extent” (4). Cronbach's alpha values for all scale items are reported in Table 2 .

*Change-oriented leadership* was measured using six items that are part of Yukl's (1999) framework of leadership styles. Examples of items are “my leader proposes new and creative ideas for improving products, services or processes” and “my leader clearly expresses what the organization can achieve or develop into”. The items were measured using a five-point scale ranging from “I strongly disagree” (1) to “I strongly agree” (5).

*Performance obstacles* were measured using items developed from the structural quality indicators included in the national system of hospital quality indicators ( NDH, 2019 ). The following four items were included in our survey: “Do you sometimes experience that patient problems are not treated because (1) the required equipment was not available? (2) the right competence was not available? (3) the organization of the work prevented it? (4) of insufficient staffing?” The items were measured using a five-point scale ranging from “no” (1) to “yes, almost every day” (5).

*Job satisfaction* was measured using three items from the Copenhagen Psychosocial Questionnaire (COPSOQ) ( Kristensen and Borg, 2001 ). The items included were “How satisfied are you with (1) your job opportunities? (2) your opportunities to use your skills? (3) your job, all things considered?”. The items were measured using a four-point scale ranging from “very dissatisfied” (1) to “very satisfied” (4).

*Participation in decision-making* was measured using the autonomy scale of the Organization Assessment Survey ( Dye, 1996 ). The following four items were included: (1) In my department, we get to influence the standards that constitute good work. (2) In my department, we often have the opportunity to influence goals or actions. (3) All employees in my department are involved in important decisions that affect them. (4) Employees have good opportunities for influence. The items were measured using a five-point scale ranging from “I strongly disagree” (1) to “I strongly agree” (5).

### Data analysis

Basic descriptive statistics, bivariate correlations and Cronbach's alpha were analysed using SPSS (2017) . Bivariate correlations were used to analyse relations between variables. Cronbach's alpha was used to assess internal consistency of factorial dimensions. AMOS ( Arbuckle, 2017 ) was used for the remaining analysis. Confirmatory factor analysis (CFA) was carried out in order to ensure the validity of measurement concepts. CFA ensures concept validity by demonstrating that the overlap with each factor is acceptable. Further, the structural model was estimated using structural equation modelling (SEM). Using SEM allowed us to evaluate the relationships between the latent factors in the hypothesized theoretical model. Bootstrap analysis (5000 bootstrapped resamples) was performed to estimate indirect effects and the mediating role of participation in decision-making ( Hayes, 2013 ).

We used the following indicators to evaluate model fit in relation to CFA and assessment of the structural model: the root mean square error of approximation (RMSEA), the Tucker–Lewis index (TLI), incremental fit index (IFI), relative fit index (RFI), normed fit index (NFI) and comparative fit index (CFI). We defined RMSEA scores of less than 0.08 ( Browne and Cudeck, 1992 ) and values of 0.90 or more for the other indicators ( Hoyle, 1995 ) as indicating good fit.

## Results

### Descriptive statistics

Descriptive statistics are presented in Table 1 . Statistical variation was considered to be satisfactory for all dimensions.

### Correlations

Correlations between the five latent factors, which were between −0.38 and 0.56, were low to moderate ( Table 2 ). Organizational change was negatively correlated with job satisfaction (−0.19) and participation in decision-making (−0.13). Organizational change was positively correlated with performance obstacles (0.22). Organizational change was negatively correlated (−0.10) with change-oriented leadership. Change-oriented leadership was negatively correlated with performance obstacles (−0.27) and positively correlated with job satisfaction (0.43) and participation in decision-making (0.56). Job satisfaction and participation in decision-making were negatively correlated with performance obstacles (−0.27 and −0.38 respectively), whereas participation in decision-making and job satisfaction were positively correlated (0.54). The correlations between job satisfaction and performance obstacles and between organizational change and change-oriented leadership were not hypothesized in our theoretical model. All other correlations were consistent with our theoretical model.

### Construct validity and internal consistency

CFA was carried out for the five latent factors and their respective indicators before testing the structural model. The latent factors were allowed to correlate in the model. The analysis indicated acceptable model fit (RMSEA = 0.06, NFI = 0.93, RFI = 0.91, IFI = 0.95, TLI = 0.94, CFI = 0.95) ( Table 3 ). Standardized factor loadings were satisfactory, ranging from 0.59 to 0.90. The internal consistency analysis shows Cronbach's alpha values ranging from 0.75 to 0.92 ( Table 2 ). The homogeneity of factors was considered to be good.

### Test of structural model

**Figure 2:**
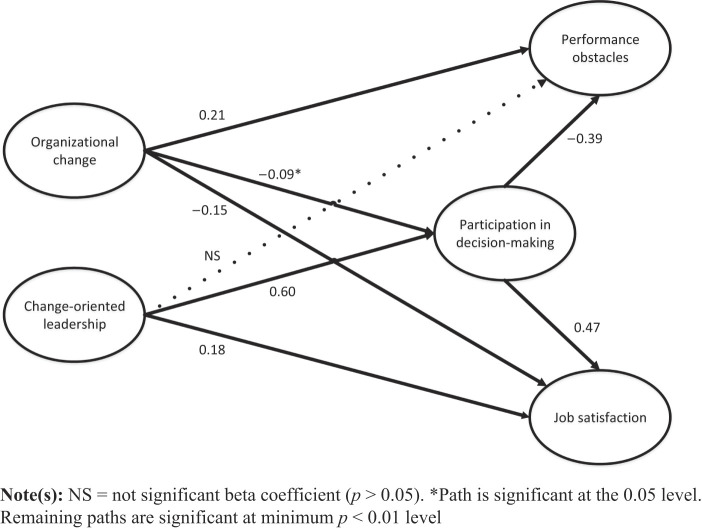
Estimated standardized path coefficients

All fit indicators were estimated within recommended thresholds supporting that the hypothesized structural model fit the data (RMSEA = 0.06, NFI = 0.93, RFI = 0.92, IFI = 0.95, TLI = 0.94, CFI = 0.95) ( Table 3 ). Moreover, estimated beta coefficients generally support the underlying theoretical model and hypotheses ( Figure 2 ), except for the relationship between change-oriented leadership and performance obstacles ( H3 ) which was found not to be significant. Organizational change was directly and positively related to performance obstacles ( *β* = 0.21), supporting H1 . Organizational change was directly and negatively related to job satisfaction ( *β* = −0.15), supporting H2 . Change-oriented leadership was directly and positively related to job satisfaction ( *β* = 0.18), supporting H4 . Organizational change was negatively related to participation in decision-making ( *β* = −0.09), and change-oriented leadership was positively related to participation in decision-making ( *β* = 0.60). Participation in decision-making was further negatively related to performance obstacles ( *β* = −0.39) and positively related to job satisfaction ( *β* = 0.47).

Results from 5000 bootstrap replications showed that the hypothesized indirect effects were supported ( H5 a–d ): organizational change → participation in decision-making → job satisfaction (standardized indirect effect = −0.04; 95% CI = −0.088,−0.003), change-oriented leadership → participation in decision-making → job satisfaction (standardized indirect effect = 0.28; 95% CI = 0.197, 0.373), organizational change → participation in decision-making → performance obstacles (standardized indirect effect = 0.03; 95% CI = 0.003, 0.077), change-oriented leadership → participation in decision-making → performance obstacles (standardized indirect effect = −0.23; 95% CI = −0.317, −0.161).

## Discussion and implications for practice

In summary, we found that the organizational changes in question were positively related to performance obstacles both directly and indirectly through participation in decision-making, meaning that more change was related to a higher prevalence of performance obstacles. Organizational change was also negatively related to job satisfaction, both directly and indirectly. Change-oriented leadership was negatively related to performance obstacles, but only indirectly through participation in decision-making, whereas it was positively related to job satisfaction both directly and indirectly.

As hospitals are subjected to increasing demands, reforms and policy changes, they have no option but to continuously change. A crucial question is whether these changes contribute to improving service quality. Former research on the effects of NPM-inspired reforms has identified disappointing results ( Braithwaite *et al.* , 2016 ). In our study, which is located at the department level, findings suggest that more change is actually related to a higher prevalence of performance obstacles. There is reason to comment on the direction of causality in this relationship. Departments that have a high prevalence of performance obstacles could be subjected to more change in order to solve these issues. However, the changes included in the data – which are changes to management, organizational structures and overall goals and strategies – are not the type of changes decided on at the level of the individual departments where our respondents perform their work. We therefore believe it is more reasonable to interpret our finding as an indication that organizational changes which in the current health policy climate are often motivated by cutting costs and increasing control and efficiency may indeed create more work system performance obstacles. This interpretation of the causal direction is also supported by the argument of change as contributing to increased job demands as well as being a job demand in and of itself and consistent with previous research which has shown that high-demand work environments for physicians have a negative impact on service quality ( Krämer *et al.* , 2016 ). The mechanisms underlying this relationship may be that job demands lead to lower work engagement and negative emotions, which in turn contribute to less extra-role performance, narrower cognitive skills and less resourceful and solution-oriented workers ( Bakker *et al.* , 2008 ; Fredrickson, 2001 ), as was described in more detail in the theory section.

A second, and equally crucial, question is how employees are affected by organizational change. Adding to an existing literature which has so far reported mixed results on the question of how organizational change in healthcare organizations impacts physician job satisfaction ( Westgaard and Winkel, 2011 ), our study finds a significant and negative relationship. Different changes may cause different employee outcomes ( Bernstrøm and Kjekshus, 2015 ), and there is a need in the literature on the effects of organizational change for studies that specify the form of change in question. Our study contributes towards this need by specifically including changes related only to management, organizational structures, overall goals and strategies and relating these changes to the wider literature on healthcare reform and physician reactions to reforms. Our findings suggest that these specific changes do in fact contribute to decreases in physician job satisfaction. This could, first, be because the changes represent job demands that are generally believed to negatively affect job satisfaction ( Alarcon, 2011 ). Secondly, considering the importance of the professional work of physicians for their job satisfaction ( Casalino and Crosson, 2015 ), because these specific changes affect the work in ways that make them less satisfied with their opportunities to perform their job in the way that they prefer.

In the terms of the JD-R framework, organizational change appears to be a job demand, whereas change-oriented leadership and participation in decision-making are job resources. Our study is not a test of a comprehensive JD-R model, but we included the job resource of participation in decision-making as a meditator because of its assumed centrality to the profession in question, because the literature on changes in the healthcare field has been concerned with how such participation is changing in healthcare systems (Byrkjeflot, 2011) and because medical engagement is called for in the literature on healthcare service improvement ( Spurgeon *et al.* , 2008 ). The test of the relationships between participation in decision-making and performance obstacles and job satisfaction resulted in some of the largest effects in our model. Our findings suggest that participation in decision-making on issues of defining success criteria, goals and actions and influence in decisions affecting the employees as well as having good opportunities for influence in general is actually quite important in relation to job satisfaction and performance obstacles. Regarding the positive relationship with job satisfaction, the underlying mechanisms could be the general positive effect that the JD-R model and conservation of resources theory of stress posit between valued job resources and job satisfaction, or, again, there could also be a more physician-specific relationship as participation in decision-making allows physicians to shape the way work is performed, thus leading to physician job satisfaction. Regarding the negative relationship with performance obstacles, we believe the mechanisms could be both the fact that job resources foster more engaged workers and that physician participation in decision-making distributes decision-making across a wide set of actors who are in intimate contact with potential workflow problems and who are also able to come up with solutions before these problems become actual obstacles.

Job resources are particularly important in fostering motivation and work engagement when job demands are high ( Bakker and Demerouti, 2007 ). This point should be noted in the case of physicians, as their work is characterized by high demands in several different categories. This means that while we found a rather weak relation between organizational changes and the job resource of participation in decision-making in the present study, the fact that a significant and negative relation was indeed found could nevertheless have substantial negative effects for the well-being and performance of physicians due to their overall high demand work situation. It should also be noted that our survey only asked respondents to report changes in the past 12 months. If the negative relationship between changes and participation in decision-making has been persistent over longer periods of time prior to our study, and continues beyond the year respondents reported on, the total effect on the job resource of participation in decision-making could be larger in a long-term perspective. If this is the case, this development runs in the opposite direction of current calls for more medical engagement in hospitals ( Spurgeon *et al.* , 2011 ).

On the contrary side, however, boosting the job resource of participation in decision-making in a high demand context is also important, and our findings suggest that change-oriented leadership may contribute to do so. Leadership has often not been included in research applying the JD-R-model ( Schaufeli, 2015 ), but the test of the model in our study suggests that change-oriented leadership may actually be considered a job resource. We believe this is a valuable finding in relation to existing literature on job demands and job resources and change-oriented leadership, as well as to managerial practice. Based on previous research, we expected change-oriented leadership to be positively related to job satisfaction and negatively related to performance obstacles. We found a relatively small direct relationship between change-oriented leadership and job satisfaction. This may be because physician job satisfaction is directly related to their professional work. There may be other leadership styles, such as forms of leadership that are not primarily managerial, but more directly related to the professional work of physicians, that have a stronger direct relationship with physician job satisfaction. We also found a non-significant relationship with performance obstacles. However, the mediation of participation in decision-making in these relationships suggests that change-oriented leadership effectively influences these outcomes via participation in decision-making. This is a contribution to the leadership literature, as the role of autonomy as a mediator of change-oriented leadership outcomes has not previously been clearly established ( Borgmann *et al.* , 2016 ). The relationship between change-oriented leadership and autonomy in the form of participation in decision-making is in fact the strongest in our model, suggesting that change-oriented middle managers are able to allow their subordinates decision latitude and include them in processes that impact their work and their work environment. Our findings support an argument for the importance of change-oriented leadership in work environments where demands are high, change is continuous and often divergent, and autonomy is highly valued by employees, important to job satisfaction and a contributor to organizational performance. Finally, while there might have been a stronger direct relationship between more purely professional leadership of physicians and job satisfaction, hospital middle managers hold large, managerial responsibilities in their leadership roles. They are expected to serve as connectors between the control and cure worlds, and we believe that identifying ways in which this leadership can be constructively executed is of value.

Our study focusses on outcomes at the department level. This is where middle managers perform their leadership, employees experience their opportunities for participation in decision-making and their job satisfaction, and performance obstacles are encountered. However, this is not the organizational level at which the types of changes we have measured are normally initiated. We did not hypothesize a relationship between change-oriented leadership and organizational change, because we do not believe it is reasonable to assume that change-oriented middle managers in hospitals meaningfully influence the initiation of changes to management, organizational structures or overall goals and strategies ( Edling and Sandberg, 2013 ). Their responsibility is rather to implement, adapt and translate top management decisions ( Williamsson *et al.* , 2016 ). There is, however, a significant and negative correlation between organizational change and change-oriented leadership in our data, and it can be interpreted in two ways. First, change-oriented middle managers may be able to buffer their department from changes initiated at higher organizational levels or able to prioritize which change initiatives to implement in their own department. In this interpretation, change-oriented leadership negatively impacts the frequency of the types of change included in our variable. Second, causation could run in the opposite direction. In this interpretation, frequent organizational changes may reduce the opportunity for middle managers to perform their leadership in a change-oriented manner. Frequent changes, particularly of the kind that stem from NPM inspired policies, could impose too many demands on these managers for them to be able to prioritize these leadership activities ( Wallin *et al.* , 2014 ). The content of these changes may also impact their decision latitude negatively, leaving them relatively more powerless in shaping a change-oriented leadership at the department level.

In conclusion, we believe it is relevant to connect our findings to the identified need for medical engagement in improving healthcare quality. The studied organizational changes appear to contribute to increased performance obstacles and decreased physician job satisfaction, while also being to some extent unavoidable due to pressures from the policy environment surrounding hospital organizations. Implementing managerial practices that offset the negative effects of these changes could enable hospitals to strike a balance between meeting the demands of new policies, maintaining a positive work environment for their physicians and avoiding obstacles to their job performance.

There are two distinct, but related, managerial implications to be drawn from our findings. First, medical engagement implies the involvement of physicians in organizational issues as well as in the professional work of treating patients. Participation in decision-making is an aspect of such engagement and should be encouraged and safeguarded by hospital leadership in change processes as well as in day-to-day operations. Opportunities for taking part in and influencing organizational decisions imply a distribution of leadership to a wider group of actors than formal managers only and a potential for bringing a more diverse set of knowledge and competencies into decisions on issues important to quality outcomes ( Denis and Baker, 2015 ). This is particularly important in highly complex organizations such as hospitals, where different actors are highly specialized within different professional fields ( Denis *et al.* , 2010 ). This constructive relationship between medical engagement and quality outcomes has been documented in previous research ( Spurgeon *et al.* , 2011 ). Our findings suggest the same effect. It could therefore be argued that in order to increase or at least maintain service quality, organizational changes should be aimed at encouraging physician participation in decision-making rather than serve to impede it. Bridging the divide between the control and cure worlds and engaging physicians in decisions on organizational issues, however, is challenging. In order for such engagement to be successful, managers who initiate changes need to understand and appreciate the identities and mindsets of physicians ( Bååthe and Norbäck, 2013 ) and translate change initiatives to operative-level physicians in ways that highlight the potential benefits to the work of professionals ( Øygarden and Mikkelsen, 2019 ).

Further, the medical engagement concept as defined by Spurgeon *et al.* (2008) implies not only participation from physicians in organizational issues, but also a recognition of this participation from the organization. This brings us to the second implication for management. Our findings suggest that giving hospital middle managers capabilities and opportunities to exercise change-oriented leadership at the department level may be a valuable strategy to fulfilling this recognition. In order to do so, they need to be equipped with appropriate leadership training, and the organizational structures surrounding their leadership positions, roles and tasks need to be evaluated and redesigned in order to create space and find time for them to be change-oriented leaders.

## Limitations and implications for future research

Our study has certain limitations. First, the cross-sectional nature of the design does not allow us to conclude on the causal direction of the tested relationships. While the theoretical framework we have used to build our hypothetical model and previous research support our arguments, a longitudinal design testing developments over time could offer more certain findings. Second, the overall response rate of 24.5% suggests that we cannot be sure that the selection of employees who responded is completely representative of all hospital physicians. Having analysed the respondents against known hospital demographics, we are not aware of any systematic biases in the sample. It may be that those who did answer skew towards being more loyal to the organization than those who did not. The sample comprises physicians working in Norwegian hospitals. However, there are similarities in the development of the healthcare sector internationally, and we believe the issues discussed in this study will be of importance in other settings as well.

Third, while the variable of organizational change is restricted to including changes that are (1) typically initiated at levels higher than the department and (2) often divergent in nature vis-à-vis a professional logic in the hospital context, the measure does not reveal specifics about who initiated the change or its specific content. There are also limitations to self-reported measures of change, as individuals experience and make sense of changes in different ways ( Rafferty and Griffin, 2006 ). Future studies including a more content-specific and objective measure of organizational change could offer valuable and more refined insights into the relationships tested in our model. Fourth, the data set did not enable us to include an outcome variable directly measuring departmental or individual performance. Future studies including such a variable would add valuable insight into the exact effects of organizational change and change-oriented leadership on hospital performance.

Finally, although we do not include other job demands, engagement or emotions as mediating variables in our model, the hypothesized relationships we present have a solid foundation in previous research on these mediators and serve to complement existing literature on the impact of organizational change on employee and performance outcomes in hospitals. The relationship between participation in decision-making and performance obstacles is among the strongest in our tested model. In order to increase our understanding of how participation in decision-making contributes towards health service quality, both quantitative studies including more mediating variables such as motivation, positive emotions or work engagement and qualitative studies of participatory processes could provide findings valuable both to the literature on medical engagement and to managerial practice.

## Figures and Tables

**Table 1:** Descriptive statistics

Dimensions	Scale	Mean	SD
Organizational change	1–4	2.19	0.71
Change-oriented leadership	1–5	3.30	0.93
Performance obstacles	1–5	2.36	0.82
Job satisfaction	1–4	3.01	0.56
Participation in decision-making	1–5	3.07	0.87

**Table 2:** Correlations among variables and Cronbach's alpha in diagonal

Dimensions	1	2	3	4	5
1. Organizational change	(0.75)				
2. Change-oriented leadership	−0.10*	(0.92)			
3. Performance obstacles	0.22**	−0.27**	(0.81)		
4. Job satisfaction	−0.19**	0.43**	−0.27**	(0.77)	
5. Participation in decision-making	−0.13**	0.56**	−0.38**	0.54**	(0.92)

**Note(s)** : ** Correlations are significant at the 0.01 level (two-tailed). * Correlations are significant at the 0.05 level (two-tailed)

**Table 3:** Model fit descriptions related to the measurement and structural model

	RMSEA	NFI	RFI	IFI	TLI	CFI	Chi-square
Measurement model	0.06	0.93	0.91	0.95	0.94	0.95	453.46
Structural model	0.06	0.93	0.92	0.95	0.94	0.95	454.71
